# Hepatitis C and HIV detection by blood RNA-sequencing in cohort of smokers

**DOI:** 10.1038/s41598-023-28156-4

**Published:** 2023-01-24

**Authors:** Jarrett D. Morrow, Peter J. Castaldi, Robert P. Chase, Jeong H. Yun, Gregory L. Kinney, Edwin K. Silverman, Craig P. Hersh

**Affiliations:** 1 Channing Division of Network Medicine, Brigham and Women’s Hospital, 181 Longwood Avenue, Boston, MA 02115 USA; 2 Division of Pulmonary and Critical Care Medicine, Brigham and Women’s Hospital, Boston, MA USA; 3 Department of Epidemiology, Colorado School of Public Health, University of Colorado Anschutz Medical Campus, Aurora, CO USA

**Keywords:** RNA sequencing, Transcriptomics, Public health, Infectious diseases, Hepatitis, HIV infections

## Abstract

Detection of viruses by RNA and DNA sequencing has improved the understanding of the human virome. We sought to identify blood viral signatures through secondary use of RNA-sequencing (RNA-seq) data in a large study cohort. The ability to reveal undiagnosed infections with public health implications among study subjects with available sequencing data could enable epidemiologic surveys and may lead to diagnosis and therapeutic interventions, leveraging existing research data in a clinical context. We detected viral RNA in peripheral blood RNA-seq data from a COPD-enriched population of current and former smokers. Correlation between viral detection and both reported infections and relevant disease outcomes was evaluated. We identified Hepatitis C virus RNA in 228 subjects and HIV RNA in 30 subjects. Overall, we observed 31 viral species, including Epstein-Barr virus and Cytomegalovirus. We observed an enrichment of Hepatitis C and HIV infections among subjects reporting liver disease and HIV infections, respectively. Higher interferon expression scores were observed in the subjects with Hepatitis C and HIV infections. Through secondary use of RNA-seq from a cohort of current and former smokers, we detected peripheral blood viral signatures. We identified HIV and Hepatitis C virus (HCV), highlighting potential public health implications for the approach described this study. We observed correlations with reported infections, chronic infection outcomes and the host transcriptomic response, providing evidence to support the validity of the approach.

## Introduction

Studies of viruses in human tissues have helped to better understand the human virome and its role in human disease^1,2^. Through secondary use of human RNA and DNA sequencing to detect viruses, evidence has emerged regarding a healthy human blood virome^3,4^. In a study of the peripheral blood DNA virome in 8,240 individuals, Moustafa and colleagues^3^ repurposed human WGS data and were able to map the WGS data to sequences of 94 different viruses, including 19 human viruses, and observed differences in virus profiles across age, sex and ancestry^3^. Human Cytomegalovirus (CMV), HHV-6A, HHV-6B, Epstein–Barr virus (EBV) and Torque Teno Virus (TTV, Anellovirus) were among those detected. In a multi-tissue study repurposing RNA sequencing (RNA-seq) data by Kumata and colleagues^4^, EBV, CMV, TTV and human papillomavirus were among the viruses observed in blood.

We hypothesize that detection of viruses known to cause long-term latent infections within existing study populations would be of particular interest, as this could enable epidemiologic surveys and perhaps lead to diagnosis and therapeutic intervention for subjects from study populations of various diseases^5^. To assess the potential impact of this question in existing study populations, we sought to detect peripheral blood viral signatures by repurposing RNA-seq data in a population of current and former smokers with and without chronic obstructive pulmonary disease (COPD).

## Results

After quality control procedures, peripheral blood RNA-seq data were available for 3,984 samples, including 1601 COPD cases and 1609 controls (Table S1 in the online supplement). Approximately a third of subjects were current smokers and twenty-five percent were African American. Also included were 218 never-smokers. Using reads not mapped to the human genome during the gene expression analysis, we detected viral RNA in the blood RNA-seq data using PathSeq (see “Methods”). Focusing on viruses with public health implications, we detected Hepatitis C virus (HCV) RNA in 228 subjects and HIV RNA in 30 subjects, defined as the mapping of at least one read to the viral genome in at least two subjects. The relaxed threshold was implemented to reduce false negatives. In total we observed 31 viral species eclipsing the detection threshold, including Epstein-Barr virus and Cytomegalovirus (Table S2 in the online supplement).

### HCV detection

A medical history of Hepatitis C infection was not obtained in COPDGene. However, 171 subjects with RNA-seq data had a self-reported history of liver disease at the 5-year follow-up and an additional 18 subjects reported liver disease at the 10-year follow-up. Of the 228 subjects with detectable HCV RNA, 77 were among the 189 total subjects with liver disease (Table 1), representing a significant enrichment (*p* < 0.0001) and suggesting high specificity. Although the 112 subjects self-reporting liver disease and lacking HCV RNA detection indicates the sensitivity may not be high, reduced HCV RNA loads would be observed in subjects undergoing treatment. Subjects with detected HCV RNA were younger with fewer comorbidities, and tended to be male, current smokers and African-American (Table 1). We did not observe a significant difference between the mapped read counts for the 77 subjects with self-reported liver disease compared with the remaining 151 (*p* value = 0.5; Mann–Whitney test).

**Table 1:** Demographics of subjects with and without detected levels of peripheral blood Hepatitis C virus (HCV) or HIV RNA.

	HCV detected (n = 228)	HCV not detected (n = 3756)	Statistical tests
Liver disease reported *	77 ^#^	112 ^##^	*p* value < 0.0001 hypergeometric test
Liver disease not reported *	151	3644	
Age (years)	59.9 ± 5.2	65.6 ± 8.9	*p* value < 0.0001 t-test
Sex (% Male/n)	67.5% / 154	49.5% / 1858	*p* value < 0.0001 Fisher's Exact Test
Race (% AA/n)	67.5% / 154	24.4% / 918	*p* value < 0.0001 Fisher's Exact Test
Current smoking—yes (%/n)	68.9% / 157	33.1% / 1245	*p* value < 0.0001 Fisher's Exact Test
Comorbidity score ** (range 0–14)	2.40 ± 1.91	2.90 ± 1.97	*p* value = 0.00013 t-test

	HIV detected (n = 30)	HIV not detected (n = 3954)	
HIV infection self-reported *	22 ^@^	83 ^@@^	*p* value < 0.0001 hypergeometric test
HIV infection not self-reported *	8	3871	
Age (years)	57.2 ± 5.1	65.3 ± 8.8	*p* value < 0.0001 t-test
Sex (% Male/n)	60% / 18	50% / 1994	*p* value = 0.36 Fisher's Exact Test
Race (% AA/n)	83% / 25	26% / 1047	*p* value < 0.0001 Fisher's Exact Test
Current smoking—yes (%/n)	70% / 21	35% / 1381	*p* value < 0.0001 Fisher's Exact Test
Comorbidity score ** (range 0–14)	2.43 ± 1.83	2.88 ± 1.97	*p* value = 0.2 t-test

^#^ 68 at 5-year follow-up and 9 at 10-year follow-up.

^##^ 103 at 5-year follow-up and 9 at 10-year follow-up.

@ 15 at 5-year follow-up.

@@ 47 at 5-year follow-up.

*At any visit in the COPDGene study.

**Sum of comorbidities reported, considering Coronary Heart disease, Diabetes, Congestive heart failure, Stroke, Osteoarthritis, Osteoporosis, Hypertension, High cholesterol, Gastroesophageal reflux disease, Stomach ulcers, Obesity, Sleep apnea, Hay fever, Peripheral Vascular Disease^30^.

**Figure 1:**
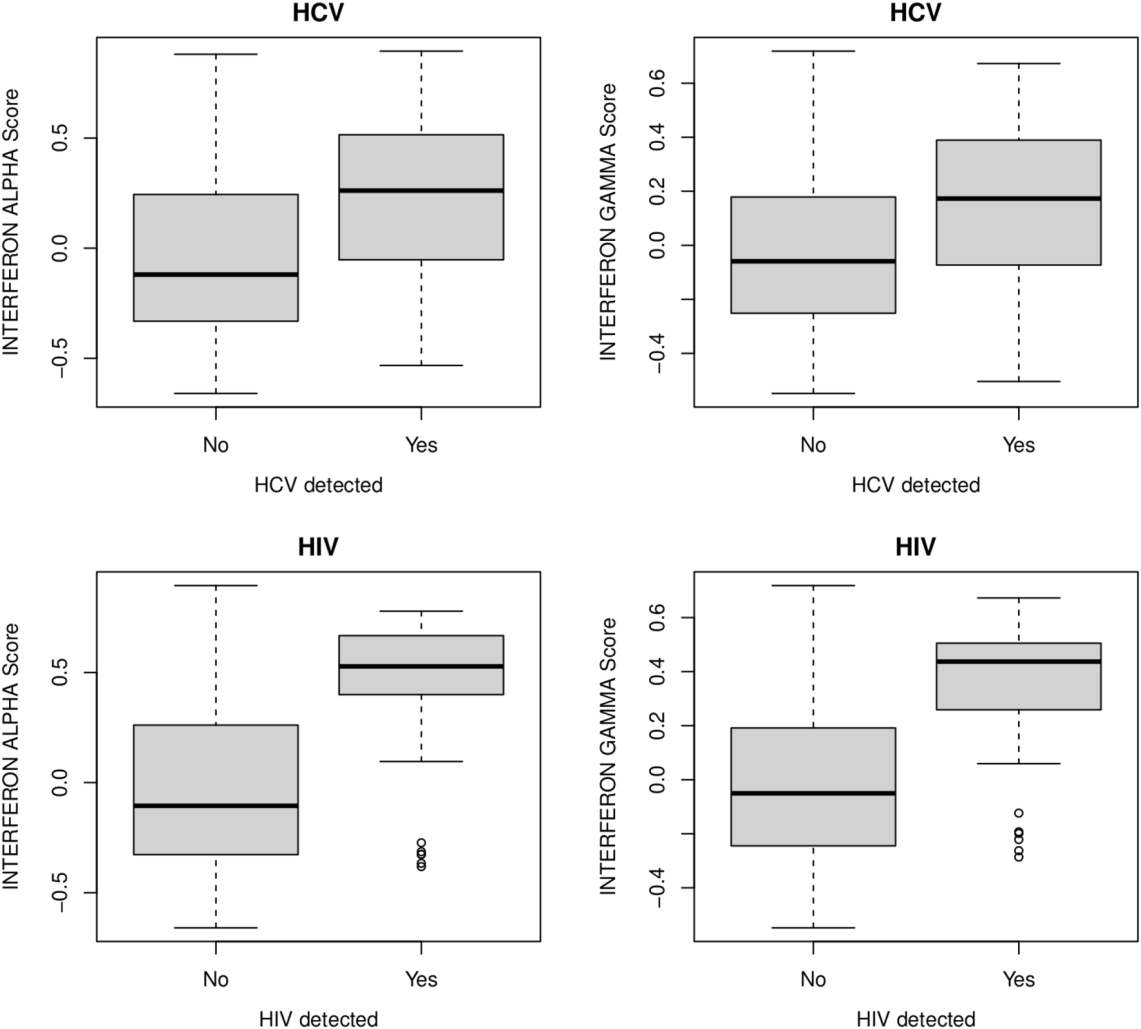
Box plots of the interferon alpha and interferon gamma transcriptomic scores calculated using gene set variation analysis (GSVA)^25^ and the MSigDB Hallmark pathway gene sets^24^ for the subjects with and without detected Hepatitis C virus (HCV) or HIV RNA.

We created interferon scores using the RNA-seq gene expression data to observe correlation between detection of infections and host response (see “Methods”)^6^. We integrated the viral detection data with the scores for the interferon alpha and interferon gamma pathways to observe score values in the subjects with HCV detection compared with the subjects lacking viral detection. We observed higher expression scores for interferon alpha and interferon gamma pathways (*p* value < 0.0001; t-test) in the subjects with HCV RNA detection (Fig. 1). We also observed significantly (*p* value < 0.0001; t-test) lower levels of a published HCV down expression score and higher levels of an HCV up expression score in the subjects with HCV detection (Fig. S1 in the online supplement), suggesting correlation between RNA detection and HCV infections.

### HIV detection

Among the subjects with RNA-seq data, 105 self-reported HIV infections from questionnaires at any COPDGene study visit. Of the 30 subjects with detectable HIV RNA, 22 were among the 105 subjects who self-reported an HIV infection (Table 1), representing a significant enrichment (*p* < 0.0001). The eight remaining subjects (Table 1) with detected HIV RNA could be either false-positive findings or undiagnosed individuals. The 83 subjects self-reporting HIV infections and not identified via RNA-seq analysis indicates the sensitivity may not be high, potentially due to therapies reducing HIV RNA loads. Although medication use data are available for 3820 of the 3984 subjects included in this study, of the 66 total subjects reporting use of medication to treat HIV 65 had also self-reported an HIV infection. The one additional subject treated with HIV medication was not among those with detectable HIV RNA. Subjects with detected HIV RNA tended to be younger, current smokers and African-American (Table 1). We did not observe a significant difference between the mapped read counts for the 22 subjects with self-reported infections compared with the remaining eight (*p* value = 0.7; Mann–Whitney test). We observed higher expression scores for interferon alpha and interferon gamma pathways (*p* value < 0.0001; t-test) in the subjects with HIV RNA detection (Fig. 1). We also observed significantly (*p* value < 0.0001; t-test) lower levels of a published HIV down expression score and higher levels of *IFI27* in the subjects with HIV detection (Fig. S1 in the online supplement), suggesting correlation between RNA detection and HIV infections. Relevant to the temporal relationship, 15 of the 22 with detectable HIV RNA first self-reported infections at the 5-year follow-up (Table 1), the blood sample collection visit. HCV RNA was detected in 20 of the 105 subjects who self-reported HIV infections and was detected in seven of the 30 subjects with detectable HIV RNA. High rates of co-occurrence of HIV and HCV are consistent with known shared risk factors for these infections^7^.

## Discussion

Through repurposing of blood RNA-seq data, we detected RNA of 31 viral species including HCV and HIV. The protocols used in the creation of the RNA-seq data in this study did not include polyA enrichment. A previous study demonstrated difficulty detecting viral RNA in polyA enriched RNA-seq data compared with non-polyA enriched RNA-seq data^8^. Although Kumata and colleagues^4^ detected some of the top viruses identified in this study, including EBV and CMV, their exclusion of HIV based on mapping specificity concerns and use of polyA enriched data from the Genotype-Tissue Expression (GTEx) project^9^ prevents a more complete comparison of findings, particularly with respect to HCV. The study of human viruses by Moustafa and colleagues^3^ involved the use of whole-genome DNA sequencing. Although we detected viral gene expression mRNA from infections caused by some DNA viruses identified in that study, such as EBV, CMV and TTV, the RNA from HCV and HIV infections would not be represented in their results to allow a direct comparison.

We observed significant enrichment of subjects with reported liver disease among those with detectable HCV RNA. We also observed a significant enrichment of subjects self-reporting HIV infections among those with detectable HIV RNA. These findings along with the elevated interferon pathway score in those with detectable RNA suggest we have identified HCV and HIV infections and a signature of the host response, given the role of interferons in response to HCV^10,11^ and HIV infections^12,13^. The concordance of virus detection and levels of the HCV and HIV host response scores from published transcriptomic studies also suggests we have observed chronic viral infections.

From 2013 to 2016, approximately 2.4 million people in the United States were living with a HCV infection^14^ and between 10 and 20% of HCV infections will lead to cirrhosis over 20–30 years^15^ with an increased risk of hepatocellular carcinoma. Direct-acting antiviral (DAA) therapy has been highly effective over the last decade^14^, highlighting the importance and benefits of HCV detection and diagnosis. In 2019, approximately 1 million people were living an HIV infection^16^. However, approximately 13% were not yet diagnosed^16^, highlighting the potential impact of HIV detection for both population surveillance and individual counseling within existing and future research study populations.

Limitations of this study include the lack of information regarding HCV infections, though an ongoing effort to return results to study subjects will provide clinical test results to allow further evaluation of the sensitivity and specificity of the RNA-seq approach. Clinically determined HIV-negatives were also lacking and prevented a full evaluation of HIV RNA detection accuracy. We also lack a validation cohort with infection-related questionnaire data. Although we are focused on secondary use of existing sequencing data, if these data do not exist for a particular study population, metagenomic approaches are available to provide more optimized viral detection^17^. The lack of control samples in this study would also be addressed in a properly designed metagenomic experiment. Although this study does not involve clinical diagnostic methods, we believe the public health implications of the viruses detected justifies the use of our approach, as it leverages existing data to enable targeted clinical interventions for study participants.

This study is the first to identify RNA signatures of HCV and HIV in the peripheral blood of a COPD-enriched population of current and former smokers through secondary use of RNA-seq data. In this study we were able to identify signatures of viral species with pathogenic and public health implications and observed correlations with reported infections, chronic infection outcomes and host transcriptomic response. Secondary mining of existing sequencing data in disease study populations using bioinformatic approaches may reveal chronic viral infections for cost-efficient epidemiologic studies and lead to additional clinical validation and therapeutic interventions where necessary.

## Methods

### Study subjects

The Genetic Epidemiology of COPD (COPDGene) study is a longitudinal cohort study that includes more than 10,000 non-Hispanic White and African American subjects enrolled at 21 centers across the United States^18^. The COPDGene cohort includes primarily current and former cigarette smokers. The five-year follow-up visit included questionnaires, spirometry, chest computed tomography scans, and collection of blood for complete blood cell counts and RNA sequencing. Subjects were at least one month removed from an acute respiratory infection. The RNA-sequencing and data processing methods were reported previously^6,19^. Briefly, paired end reads with nominal 75 bp length were generated on an Illumina HiSeq 2500 flow cell. Sequencing was performed to an average depth of 20 million reads. For the human gene expression analyses, STAR aligner^20^ was used to map the reads to GRCH38 and RSubreads produced gene-level counts^21^ with Ensembl gene annotation^22^. We confirmed concordance between sex-specific expression features and reported sex, and concordance between variants called from RNA sequencing reads and corresponding DNA genotyping.

### Viral RNA detection

Using reads that were not mapped to the human genome during the gene expression analysis, we detected viral signatures in the COPDGene 5-year follow-up whole blood RNA-seq data using PathSeq, the microbial detection pipeline from the Genome Analysis Toolkit (GATK4)^23^, as described in a previous microbiome study^6^. Filtering of the unmapped reads was performed using PathSeq and the host reference in the GATK Resource Bundle^23^. The filtering step helps to addresses quality, host contamination or repetitive sequence issues. We then mapped the cleaned read data to viral reference genomes using PathSeq. The viral genomic reference was created using representative genomes (12,148 genomic entries) from the National Center for Biotechnology Information (NCBI). Taxonomy information for the viral genomic data was also obtained from NCBI (RefSeq-release95.catalog.gz). PathSeq output included the mapped read counts for each sample and viral species.

### Transcriptomic scores

To observe transcriptomic signatures of the host response to the detected infections, we projected the RNA-seq gene expression data onto the Hallmark pathway gene set collection from MSigDB^24^ using gene set variation analysis via the R package GSVA^25^, as previously described^6^. We focused on the Hallmark interferon alpha and interferon gamma pathway gene sets. This method creates a composite transcriptomic expression score for the set of genes within each of the pathways. We also created scores using genes observed to be differentially expressed in the blood of HCV and HIV infected individuals. In a study of peripheral blood mononuclear cells (PBMCs), Bolen and colleagues^26^ identified 109 genes differentially expressed in HCV patients relative to uninfected controls. Using GSVA and the 56 genes down-expressed in HCV and 53 genes up-expressed, we created HCV down expression and up expression scores. For HIV, we used the set of 10 genes identified by Ockenhouse and colleagues^27^, observed to be down expressed in the PBMCs of HIV seropositive individuals and differentiating seropositive from seronegative, to create an HIV down expression score. Previous studies showed higher expression of the gene *IFI27* in the peripheral blood of HIV infected individuals compared with seronegative controls^28,29^; we also analyzed expression of this gene to provide evidence of HIV infections. For this analysis, RNA-seq data were retained for genes with variance in the upper 80th percentile and for genes with average read counts greater than five. GSVA output provides expression pathway scores for each subject and gene set.

### Ethics statement

All subjects in this study provided written consent for study procedures, including genetic analysis. COPDGene was approved by the Institutional Review Boards at all participating centers. The research methods were carried out in accordance with the relevant guidelines.

### Ethics approval and consent to participate

All subjects in this study provided written informed consent. COPDGene was approved by the Institutional Review Boards at all participating centers.

**Table:** 

Clinical center	Institution title	Protocol number
National Jewish Health	National Jewish IRB	HS-1883a
Brigham and Women’s Hospital	Partners Human Research Committee	2007-P-000554/2; BWH
Baylor College of Medicine	Institutional Review Board for Baylor College of Medicine and Affiliated Hospitals	H-22209
Michael E. DeBakey VAMC	Institutional Review Board for Baylor College of Medicine and Affiliated Hospitals	H-22202
Columbia University Medical Center	Columbia University Medical Center IRB	IRB-AAAC9324
Duke University Medical Center	The Duke University Health System Institutional Review Board for Clinical Investigations (DUHS IRB)	Pro00004464
Johns Hopkins University	Johns Hopkins Medicine Institutional Review Boards (JHM IRB)	NA_00011524
Los Angeles Biomedical Research Institute	The John F. Wolf, MD Human Subjects Committee of Harbor-UCLA Medical Center	12756-01
Morehouse School of Medicine	Morehouse School of Medicine Institutional Review Board	07-1029
Temple University	Temple University Office for Human Subjects Protections Institutional Review Board	11369
University of Alabama at Birmingham	The University of Alabama at Birmingham Institutional Review Board for Human Use	FO70712014
University of California, San Diego	University of California, San Diego Human Research Protections Program	070876
University of Iowa	The University of Iowa Human Subjects Office	200710717
Ann Arbor VA	VA Ann Arbor Healthcare System IRB	PCC 2008-110732
University of Minnesota	University of Minnesota Research Subjects’ Protection Programs (RSPP)	0801M24949
University of Pittsburgh	University of Pittsburgh Institutional Review Board	PRO07120059
University of Texas Health Sciences Center at San Antonio	UT Health Science Center San Antonio Institutional Review Board	HSC20070644H
Health Partners Research Foundation	Health Partners Research Foundation Institutional Review Board	07-127
University of Michigan	Medical School Institutional Review Board (IRBMED)	HUM00014973
Minneapolis VA Medical Center	Minneapolis VAMC IRB	4128-A
Reliant Medical Group	Institutional Review Board/Research Review Committee Saint Vincent Hospital – Reliant Medical Group – Fallon Community Health Plan	1143

## Supplementary Information

Supplementary Information.

## Data Availability

Phenotype and the RNA sequencing data are available in dbGaP, accessions phs000179 and phs000765. https://www.ncbi.nlm.nih.gov/projects/gap/cgi-bin/study.cgi?study_id=phs000179.v6.p2. https://www.ncbi.nlm.nih.gov/projects/gap/cgi-bin/study.cgi?study_id=phs000765.v3.p2.

## Abbreviations

COPD: Chronic obstructive pulmonary disease
COPDGene: Genetic epidemiology of COPD
GATK: Genome analysis toolkit
GOLD: Global initiative for chronic obstructive lung disease
GSVA: Gene set variation analysis
HIV: Human immunodeficiency virus
HCV: Hepatitis C virus
MSigDB: Molecular signatures database
NCBI: National center for biotechnology information
