# Supplementary material for: Hepatitis C and HIV detection by blood RNA-sequencing in cohort of smokers
Source: Sci Rep. 2023 Jan 24;13:1357. doi: 10.1038/s41598-023-28156-4 (PMC9873751; doi:10.1038/s41598-023-28156-4)
Supplement: Supplementary file 1 — Supplementary Information. [file 41598_2023_28156_MOESM1_ESM.pdf]

Hepatitis C and HIV detection by blood RNA-sequencing in cohort of smokers

Jarrett D. Morrow[1], Peter J. Castaldi[1], Robert P. Chase[1], Jeong H. Yun[1,2], Gregory L. Kinney[3]
Edwin K. Silverman[1,2], Craig P. Hersh[1,2]

1. Channing Division of Network Medicine, Brigham and Women's Hospital, Boston, MA
2. Division of Pulmonary and Critical Care Medicine, Brigham and Women's Hospital, Boston, MA
3. Department of Epidemiology, Colorado School of Public Health, University of Colorado Anschutz Medical Campus, Aurora, CO

Figures

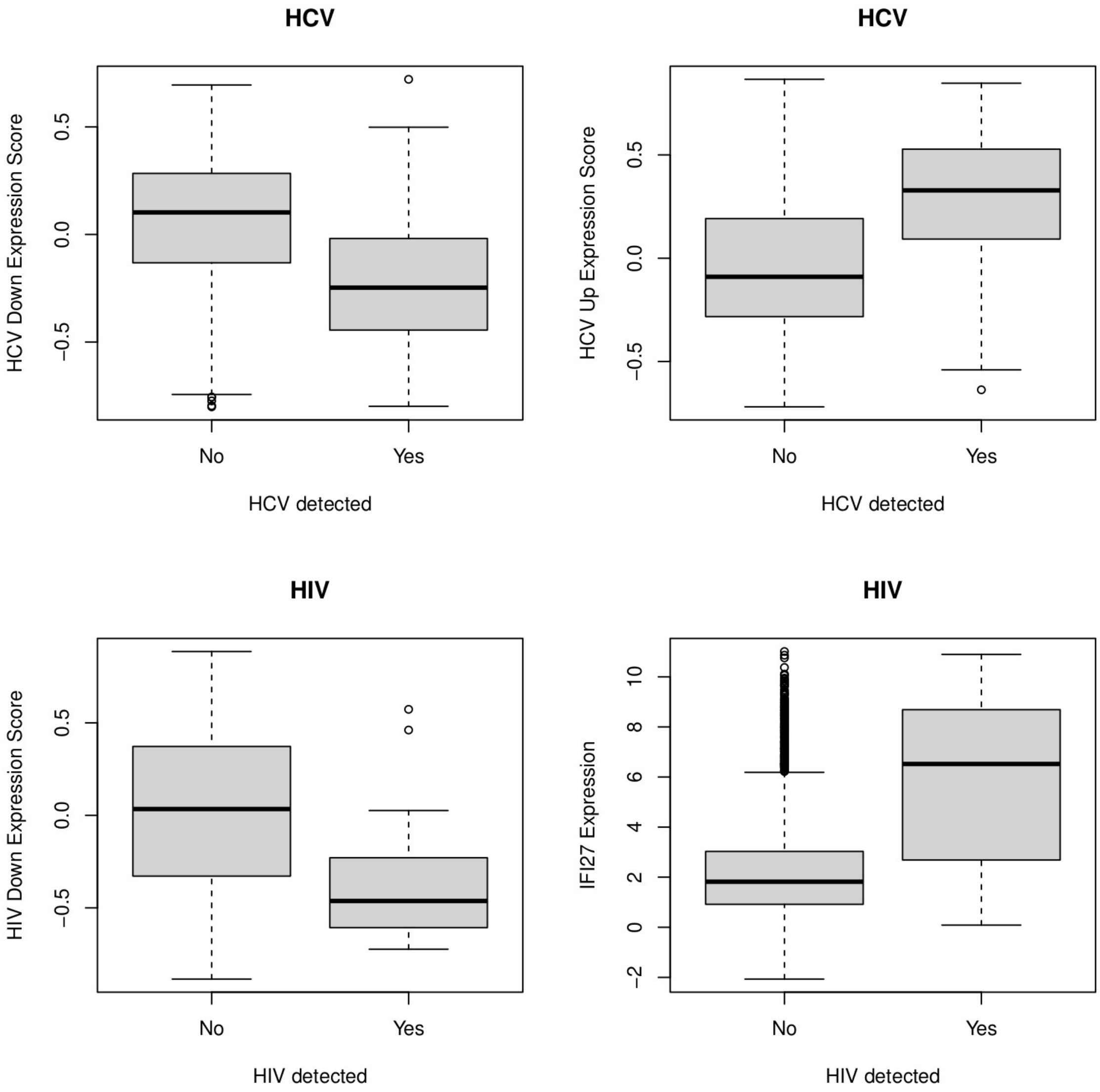

Figure S1. Box plots of transcriptomic signatures for the subjects with and without detected Hepatitis C virus (HCV) or HIV RNA. The three transcriptomic scores were calculated using gene set variation analysis (GSVA) [1] and published gene sets from differential gene expression analysis of HCV in peripheral blood mononuclear cells (PBMCs) [2] and differential expression analysis of HIV in PBMCs [3]. IFI27 gene expression was previously observed to be up-regulated in HIV peripheral blood [4,5].

Tables

Table S1. COPDGene study subjects

Demographics (N = 3984)	Mean ± sd or distribution
Age, years (n = 3961)	65.3 ± 8.8
Sex (Female / Male) (n = 3961)	1949 / 2012
Race (Non-Hispanic White / African American) (n = 3961)	2889 / 1072
Current smoking status (yes / no) (n = 3944)	1402 / 2542
Smoking status (ever / never) (n = 3961)	3743 / 218
Smoking History, pack-years (n = 3945)	41.5 ± 25.6
GOLD stage (n = 3897)<br>4<br>3<br>2<br>1<br>Control<br>PRISm *	 150<br>364<br>713<br>374<br>1609<br>481
FEV_1 % predicted (n = 3897)	79.9 ± 24.6
FEV_1 / FVC (n = 3896)	0.68 ± 0.15
Percent emphysema at -950HU (n = 3664)	5.1 ± 8.8
Body mass index kg/m^2 (n = 3948)	28.8 ± 6.2
Severe exacerbation in the year prior ** (no / yes) (n = 3945)	3611 / 334
Treated with chronic oral corticosteroids (no / yes) (n = 3888)	3834 / 54
Comorbidity score *** (range 0 to 14) (n = 3946)	2.87 ± 1.97
HIV infection (first self report)<br>Baseline<br>5-year follow-up<br>10-year follow-up	 40<br>62<br>3
Liver Disease (first self report)<br>Baseline<br>5-year follow-up (n = 3947)<br>10-year follow-up (n = 1354)	 N/A<br>171<br>18

All subjects in this study provided written consent for study procedures, including genetic analysis.

COPDGene was approved by the Institutional Review Boards at all participating centers.

Abbreviations: GOLD=Global Initiative for Chronic Obstructive Lung Disease; FEV1=forced expiratory volume in 1 sec; FVC= forced vital capacity; Control subjects have normal spirometry (FEV1% predicted ≥ 80% and FEV1/FVC ≥ 0.7); HIV = Human Immunodeficiency Virus; N/A = not available

* PRISm = Preserved Ratio Impaired Spirometry (FEV1<80% predicted with FEV1/FVC≥0.7) [6]
** Emergency department or hospital admission
*** Sum of comorbidities reported, considering Coronary Heart disease, Diabetes, Congestive heart failure, Stroke, Osteoarthritis, Osteoporosis, Hypertension, High cholesterol, Gastroesophageal reflux disease, Stomach ulcers, Obesity, Sleep apnea, Hay fever, Peripheral Vascular Disease [7].

Table S2. Viruses detected in the peripheral blood RNA-seq data from COPDGene 5-year follow-up visit

Species	Subjects with at least one read	Min read count for detected	Max read count for detected	Mean read count for detected
Human gammaherpesvirus 4 – Epstein-Barr virus (EBV)	298	1	60	4
Hepacivirus C – Hepatitis C virus (HCV)	228	1	6065	249
Pegivirus C	96	1	7528	1442
Torque teno viruses *	48	1	135	7
Pegivirus H	31	1	5788	580
Human immunodeficiency virus 1 (HIV)	30	1	66	7
Human betaherpesvirus 6B	19	1	169	12
Human endogenous retrovirus K	12	1	1	1
Human betaherpesvirus 5 - Cytomegalovirus (HCMV)	12	1	37	4
Human betaherpesvirus 6A	8	1	13	6
Hepatitis B virus (HBV)	3	19	514	264
Human gammaherpesvirus 8 - Kaposi's sarcoma-associated herpesvirus	2	2	4	3
Human mastadenovirus C	2	2	2	2
Human coronavirus 229E	2	2	38	20
Torque teno midi viruses **	2	4	4	4

* Torque teno viruses entry includes reads from 14 Torque teno species
** Torque teno midi viruses entry includes reads from 4 Torque teno midi species

References

1. Hänzelmann, S., Castelo, R. & Guinney, J. GSVA: gene set variation analysis for microarray and RNA-Seq data. *BMC Bioinformatics* **14**, 7 (2013).
2. Bolen, C. R. *et al.* The Blood Transcriptional Signature of Chronic Hepatitis C Virus Is Consistent with an Ongoing Interferon-Mediated Antiviral Response. *J. Interferon Cytokine Res.* **33**, 15–23 (2013).
3. Ockenhouse, C. F., Bernstein, W. B., Wang, Z. & Vahey, M. T. Functional Genomic Relationships in HIV-1 Disease Revealed by Gene-Expression Profiling of Primary Human Peripheral Blood Mononuclear Cells. *J. Infect. Dis.* **191**, 2064–2074 (2005).
4. Palm, A. A. *et al.* Interferon Alpha-Inducible Protein 27 Expression Is Linked to Disease Severity in Chronic Infection of Both HIV-1 and HIV-2. *Front. Virol.* **2**, (2022).
5. Huang, H. *et al.* IFI27 is a potential therapeutic target for HIV infection. *Ann. Med.* **54**, 314–325 (2022).
6. William W. Stringer, M. D. *et al.* Physiologic Insights from the COPD Genetic Epidemiology Study. *Chronic Obstr. Pulm. Dis. COPD Found.* **6**, 256–266.
7. Putcha, N. *et al.* A Simplified Score to Quantify Comorbidity in COPD. *PLOS ONE* **9**, e114438 (2014).
